# Structural Modulation of Musky Octopus Proteins by pH and Ultrasound: From Aggregates to Protein–Quercetin Emulsion Stabilisers

**DOI:** 10.3390/molecules30234570

**Published:** 2025-11-27

**Authors:** María Carmen Gómez-Guillén, Ailén Alemán, Ignacio Boto, Johana López-Polo, María Pilar Montero

**Affiliations:** 1 Institute of Food Science, Technology and Nutrition (ICTAN-CSIC), José Antonio Novais, 6, 28040 Madrid, Spain; ailen@ictan.csic.es (A.A.); mpmontero@ictan.csic.es (M.P.M.); 2 Institute of Nutrition and Food Technology (INTA), University of Chile, El Líbano, 5524, Santiago 783090, Chile; johana.lopez@inta.uchile.cl

**Keywords:** *Eledone moschata*, isoelectric precipitation, ultrasonication, myofibrillar protein-quercetin nanoparticles, Pickering emulsion, low-fat

## Abstract

This study investigates the potential of an undervalued cephalopod species, *Eledone moschata*, for producing a freeze-dried protein concentrate via acid solubilisation and isoelectric precipitation. Although nutritionally rich, the processing route significantly affected the aggregation state of the recovered proteins, as demonstrated by differential scanning calorimetry (DSC) and SDS–PAGE electrophoresis. We systematically examined pretreatments of the lyophilised protein concentrate (PC) by dispersing it across a pH range (2–10) and applying ultrasonication (US), characterising the resulting aggregates in terms of protein solubility, surface hydrophobicity, dynamic light scattering (DLS), and ζ-potential. Subsequently, ultrasound-treated protein dispersions at different pH values were used to produce protein–quercetin nanoparticles (PQ), which were analysed for particle size (DLS), yield, and quercetin entrapment efficiency. PQ dispersions at pH 2, 4, and 7 were evaluated as stabilising agents in US-treated sunflower oil emulsions containing 10% oil and were characterised by rheological properties, microstructure, and DLS particle sizing. Confocal laser scanning microscopy (CLSM) revealed that nanoparticles at pH 2 produced small, uniformly distributed fat droplets with a particle diameter of 1.5 μm. This study provides insights into how processing conditions modulate the structural and interfacial behaviour of cephalopod proteins and highlights their potential application in designing low-fat, fluid emulsions for innovative food formulations.

## 1. Introduction

The valorisation of marine animal by-products and underexploited species has gained increasing attention in recent years as part of a global effort to promote sustainable resource utilisation and reduce food waste. These undervalued materials can be transformed into value-added functional ingredients for food, nutraceutical and pharmaceutical applications [1]. Among them, cephalopods such as the musky octopus (*Eledone moschata*)—a species of limited commercial value often discarded or underutilised—represent a promising source of high-value biomolecules [2].

Proteins derived from cephalopods and other marine organisms are particularly attractive due to their high nutritional value and superior functional properties compared with conventional animal and plant proteins [3,4]. However, their functional performance is strongly influenced by processing history. Factors such as pH and physical treatments significantly affect solubility, protein profiles and surface properties [5,6]. Acid solubilisation and isoelectric precipitation are widely used to recover fish and squid myofibrillar proteins with high purity; however, processing conditions can alter protein conformation and functionality [7,8].

High-intensity ultrasonication has gained recognition as a clean and controllable technology for tailoring protein structure and functionality. The cavitation generated by ultrasound can induce partial unfolding, expose hydrophobic patches, reduce particle size and modify thermal behaviour—often improving solubility, surface hydrophobicity and interfacial activity [9,10,11,12]. These ultrasound-mediated changes enhance protein interactions with hydrophobic compounds, facilitating their assembly into nanoscale delivery systems and improving emulsifying and encapsulating properties—key for developing advanced food colloids such as Pickering emulsions [13].

Protein concentrates obtained from cephalopods, such as the mantle of the giant squid [14] or squid ovary [10], have demonstrated enhanced functional properties following ultrasonication treatment. Similarly, myofibrillar protein extracted from Asian sea bass and treated with high-intensity ultrasound exhibited strong emulsifying properties [15]. The pH of the dispersion medium also plays a critical role in conditioning marine protein behaviour, as pH-dependent dissociation of myofibrillar components affects molecular conformation, charge distribution and aggregation [16]. Moreover, the ability of marine-derived proteins to form stable complexes with phenolic compounds has been shown to enhance their emulsifying properties [4,17]. Therefore, combining pH adjustment with ultrasound pretreatment and interaction with phenolic compounds presents a promising strategy for controlling protein assembly and complexation behaviour, enabling the development of functional nanostructures from undervalued marine animal proteins for food and nutraceutical applications.

Pickering emulsions, stabilised by solid colloidal particles rather than conventional surfactants, have attracted significant attention as an alternative strategy for formulating surfactant-free or low-fat systems with enhanced physical stability, controlled microstructure and clean-label appeal [18,19]. These emulsions offer a robust platform for delivering lipophilic bioactives, particularly when protein-based particles are employed. Their incorporation can simultaneously stabilise the oil–water interface and impart added nutritional and biological functionality [20,21,22]. Among bioactive compounds, flavonoids such as quercetin are of particular interest due to their potent antioxidant and anti-inflammatory properties. However, quercetin’s poor water solubility and limited bioavailability pose challenges for direct incorporation into food and beverage matrices [23]. Entrapment into protein-based nanoparticles has emerged as an effective strategy to enhance its dispersibility, protect it from degradation and improve its functional stability and bioavailability [24]. The physicochemical stability of quercetin has been shown to improve when complexed with plant-based proteins such as zein and soy protein isolate (SPI) [25,26]. Lin et al. [27] demonstrated that entrapping quercetin in nanoparticles made from high-intensity ultrasound-treated soy protein isolate significantly enhanced its solubility and bioaccessibility. However, research on the use of marine myofibrillar proteins for producing protein–quercetin nanoparticles remains unexplored. The integration of quercetin into undervalued marine protein-based low-fat Pickering emulsions thus represents a promising, sustainable approach for developing low-calorie functional food formulations with enhanced nutraceutical potential.

The aim of this study was to prepare protein–quercetin nanoparticles (PQ) using a freeze-dried protein concentrate obtained by acid solubilisation and isoelectric precipitation, derived from the musky octopus (*Eledone moschata*). Prior to protein–quercetin complexation, the effects of varying pH conditions during protein dispersion, followed by ultrasonication, were evaluated in terms of protein solubility, electrophoretic profile, hydrophobicity and surface charge. Based on particle characteristics, yield and quercetin entrapment efficiency, acid-derived PQ nanoparticle dispersions were selected for the formulation of low-internal-phase oil-in-water (O/W) Pickering emulsions, which were subsequently characterised in terms of rheological behaviour, particle size and microstructure.

## 2. Results and Discussion

### 2.1. Properties of the Protein Concentrate

**Figure 1:**
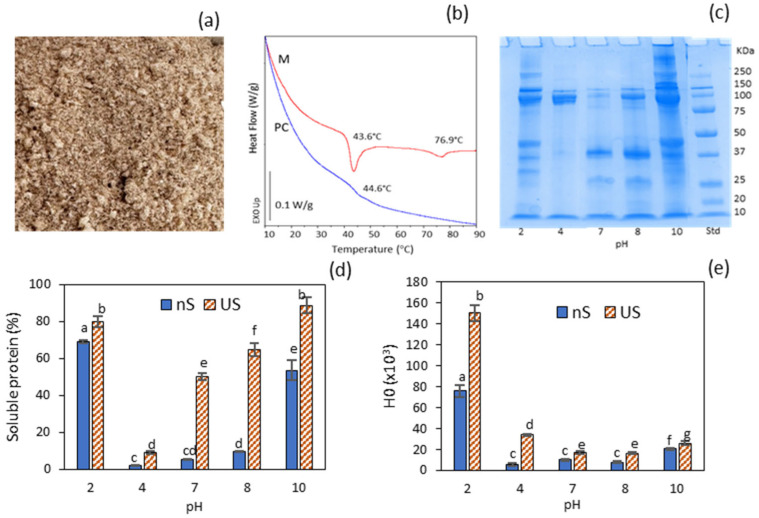
Properties of the protein concentrate (PC): (**a**) visual appearance of the dry powder; (**b**) DSC traces of the minced muscle (M) and the derived protein concentrate (PC); (**c**) electrophoretic profile (SDS–PAGE) of soluble protein from PC dispersions adjusted to different pH levels; (**d**) soluble protein of PC dispersions adjusted to different pH levels, without (nS) and with subsequent ultrasonication (US); (**e**) hydrophobicity of PC dispersions adjusted to different pH levels without (nS) and with subsequent ultrasonication (US). Different letters (a, b, c…) indicate significant differences (*p* ≤ 0.05).

The proximate composition of *E. moschata* mince (M) and protein concentrate (PC) is presented in Table 1. The protein, lipid, moisture and ash contents of M were comparable to those previously reported for the same species caught in different seasons [28]. The protein concentrate (PC), obtained via freeze-drying, exhibited a high protein content (89.6%), alongside low levels of ash (3.0%) and fat (4.9%). The low residual moisture observed in PC indicates that lyophilisation was successfully performed, resulting in a fine powder (Figure 1a). The yield of dehydrated protein concentrate relative to the initial weight of chopped octopus was 7% (41% on a dry basis). Yield reductions are likely attributable to the gradual loss of homogenised material during successive transfers, particularly during the removal of remnants of skins and tunics, which accounted for 2.4% of the initial mass. It should be emphasised that these findings provide only a preliminary indication of the system’s potential in industrially relevant contexts. Further optimisation will be required to enable practical industrial implementation.

Glutamic acid and aspartic acid were the predominant amino acid constituents in the PC, followed by arginine, leucine and lysine (Table 2). Hydrophobic amino acid residues accounted for 40.7% of the total protein. The detection of hydroxyproline residues in the protein concentrate suggests the presence of residual collagen [29], primarily originating from remaining connective tissue in tunics and tentacle suckers. Essential amino acids, excluding tryptophan (which was not determined), represented 39.51%. The nutritional score values markedly exceeded the FAO/WHO reference standards (Table 2), underscoring the protein concentrate as a high-quality source [30].

The DSC thermogram of the mince (M) exhibited two main endothermic transitions at T_peak_ = 43.6 °C (∆H = 1.24 J/g), typically attributed to myosin, and T_peak_ = 76.9 °C (∆H = 0.35 J/g), corresponding to actin (Figure 1b). This range of denaturation temperatures has also been reported for mantle proteins of other cephalopod species, such as *Dosidicus gigas* [31]. In contrast, the DSC thermogram of the protein concentrate (PC) revealed only a subtle thermal transition at 44.6 °C, indicating that both myosin and actin were extensively denatured. Freezing the precipitated protein at its isoelectric point, followed by freeze-drying, induced a highly aggregated state of the major myofibrillar proteins in the resulting PC, which may compromise its functional properties due to markedly reduced solubility. This issue can be mitigated by subjecting the protein to pH variation and subsequent ultrasonication.

### 2.2. Water Solubility and Effect of Ultrasonication

The water solubility of the protein concentrate (PC) was evaluated across a range of pH values. The SDS–PAGE profile revealed a greater diversity of bands at pH 10 and pH 2 (Figure 1c), consistent with the markedly enhanced solubility observed under these conditions (nS soluble protein fraction, Figure 1d). Notably, the most alkaline condition, which resulted in slightly lower protein solubility than at pH 2, exhibited a protein aggregation band > 250 kDa that was absent at pH 2. The band corresponding to the myosin heavy chain (MHC, ~250 kDa) appeared only at pH 10 and 2, while actin (~37 kDa) was particularly abundant at pH 7 and 8. The lowest solubility was observed at pH 4, which is closest to the isoelectric point (~5.4), where myofibrillar proteins possess a net zero charge in solution. The limited solubility at pH 7–8 (<10%, Figure 1d), coupled with the absence of the MHC band in the electrophoretic profile, was consistent with the DSC thermogram of the PC, which indicated a predominantly aggregated protein conformation. In particular, the low solubility at pH 7 (5.25%) aligns with values previously reported for pH-shifted fish protein isolates obtained via acid extraction and subsequently dried at their isoelectric point (pH 5.5) [32]. A prominent band at ~100 kDa was observed at all pH levels studied, except at pH 7. This band may correspond to paramyosin, a myofibrillar protein characteristic of invertebrates such as squid and other cephalopods [33]. However, this molecular weight could also indicate the presence of collagen single α-chains, which can be extracted under both acidic and alkaline conditions [29]. Notably, two prominent ~100 kDa split bands detected at pH 4—coinciding with the absence of both MHC and actin bands—suggested a predominant collagen presence (α1 and α2 chains) in this sample. This interpretation was supported by the detection of hydroxyproline in the protein concentrate (PC).

### 2.3. Effect of Ultrasonication

Figure 1d,e illustrate the effects of ultrasonication under varying pH conditions on the solubility and surface hydrophobicity of the protein concentrate (PC). Ultrasonication significantly enhanced protein solubility across all tested pH levels (*p* ≤ 0.05). This behaviour was attributed to protein unfolding caused by the formation, growth and subsequent collapse of bubbles generated by high-intensity ultrasonic cavitation [9]. Increases in protein solubility following ultrasonication have previously been reported for various types of proteins, including globular pea proteins [11] and myofibrillar proteins from Asian sea bass [15]. This enhancement is attributed to protein dissociation and the disruption of large, stable macro-aggregates into smaller aggregates via cavitation.

The pH condition that resulted in the highest (*p* ≤ 0.05) protein surface hydrophobicity (H_0_) was pH 2 (Figure 1e), indicating greater exposure of hydrophobic amino acid residues due to a higher degree of protein unfolding. H_0_ values at pH 10 were noticeably lower than those at pH 2. Elevated hydrophobicity values in fish and squid proteins at pH 2, compared to neutral and alkaline pH, have been previously documented [8]. Under strongly alkaline conditions (pH 10), the formation of small protein aggregates (>250 kDa) was observed in the SDS–PAGE profile. Although this slightly reduced protein solubility, it markedly decreased surface hydrophobicity, likely due to structural rearrangements and increased exposure of hydrophilic regions. Notably, the acidic ultrasonicated dispersions at pH 2 and 4 exhibited the greatest relative increases in surface hydrophobicity (H_0_) compared to their non-sonicated counterparts.

### 2.4. Effect of Quercetin Addition

**Figure 2:**
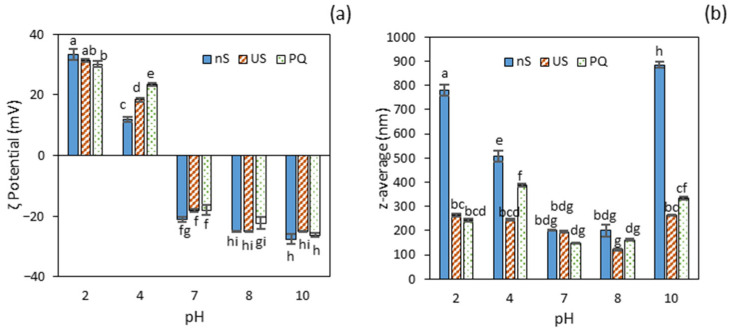
Particle properties of protein aggregates without (nS) and with subsequent ultrasonication (US), and protein–quercetin nanoparticles (PQ): (**a**) ζ potential; (**b**) average particle size. Different letters (a, b, c…) indicate significant differences (*p* ≤ 0.05).

The effect of pH, ultrasonication (US) and quercetin addition on ζ potential and particle size of protein aggregates is shown in Figure 2. The ζ potential values gradually shifted from electropositive at acidic pH (2 and 4) to electronegative between pH 7 and 10 across all samples (Figure 2a). This characteristic pH-dependent pattern primarily results from the progressive protonation of protein carboxyl groups at acidic pH and the deprotonation of amino groups under alkaline conditions. The highest net ζ potential values (*p* ≤ 0.05) were observed at the most acidic and alkaline pH levels, reflecting maximum colloidal stability and corresponding to the greatest electrostatic repulsion among protein molecules [9].

Within the acidic range, a significant reduction in ζ potential (*p* ≤ 0.05) was observed between pH 2 and 4, with a more pronounced decrease in the non-sonicated protein concentrate (nS). This behaviour is consistent with substantial myofibrillar protein aggregation in this sample, as evidenced by the marked pH-induced decline in both protein solubility and surface hydrophobicity. Ultrasound treatment had no significant effect (*p* > 0.05) on the ζ potential of the PC, except at pH 4, where a notable increase was observed compared to the non-ultrasound-treated sample. This suggests that US was effective in promoting increased electrostatic repulsion when the protein was strongly aggregated near the isoelectric point. Similarly, the addition of quercetin to the ultrasonicated protein concentrate (PQ) did not induce changes across the tested pH levels, except at pH 4, where the ultrasound induced a two-fold increase in ζ potential, possibly facilitated by enhanced surface hydrophobicity of the protein.

The particle size of untreated protein aggregates (nS) was greatest at the extreme pH values of 2 and 10 (Figure 2b), which also corresponded to the highest concentrations of soluble protein. More specifically, the protein aggregates at pH 10 were significantly larger (*p* ≤ 0.05) than those at pH 2, a finding that aligns with the observed reduction in solubility. Ultrasound (US) treatment significantly reduced (*p* ≤ 0.05) particle size, yielding protein nanoaggregates smaller than 265 nm across all tested pH levels, except at pH 7 and 8, where the size remained unchanged (*p* > 0.05) (Figure 2b). A similar average particle size (~271 nm) was previously reported for myofibrillar protein extracted from chicken breast and treated with high-intensity ultrasound at pH 6 for 15 min [9].

A reduction in particle size has also been observed in various squid proteins [10,14], attributed to the dissociation of large, aggregated protein particles into smaller ones. At pH 7 and 8, the small initial particle size (200 nm) in the non-sonicated samples (nS) was likely due to the removal of large protein aggregates during centrifugation, consistent with the low proportion of soluble protein in these samples (Figure 1). This effect, however, was not observed at pH 4, where water solubility was even lower than at pH 7–8. A possible explanation may lie in the distinct protein profile observed by SDS-PAGE in this sample, where the predominance of acid-soluble collagen (single α-chains) resulted in the retention of larger aggregates in the supernatant.

Following quercetin addition, the mean particle size of protein–quercetin nanocomplexes (PQ) varied significantly (*p* ≤ 0.05) among the samples, with the smallest size observed at pH 7 and 8 (≈155 nm) and the largest at pH 4 (389 nm). Compared with the US-treated protein dispersions, the most pronounced effect was observed at pH 4, where particle size increased by 59% upon quercetin addition. A 30% reduction in size was reported for BSA nanoparticles after quercetin loading [34], while no significant change was observed for zein or SPI nanoparticles [25,27]. In contrast, Wu et al. [35] reported a significant increase in SPI–quercetin nanoparticle size with varying quercetin concentrations. This divergence in reported outcomes suggests considerable variability depending on the type of protein, quercetin concentration, and/or preparation conditions.

The particle yield and quercetin entrapment efficiency (EE) of PQ at different pH values are presented in Table 3. Dispersions at pH 7 and 8 exhibited by far the lowest yields, suggesting that a substantial fraction of large protein aggregates was removed in the pellet following centrifugation, thereby contributing to the smaller particle sizes observed in these samples. In contrast, dispersions at pH 2 and 10 exhibited notably high yields (≥92%), whereas pH 4 showed intermediate values (72%). The EE was highest at pH 2 and pH 10 (81–82%), corresponding to the pH levels of maximum protein solubility and highest particle yields. Under these conditions, protein chains are extensively unfolded and thus more prone to interact with the flavonoid via non-covalent interactions [13].

It should also be considered that, under highly alkaline conditions, quercetin may undergo oxidation, and covalent interactions should not be disregarded [36]. These results are comparable to those reported for quercetin complexed with US-treated SPI at pH 7 (75%) [27]. Notably, despite the relatively high particle yield and larger particle size observed at pH 4, the EE was significantly lower than that at pH 7, which exhibited both the lowest yield and the smallest particle size. This suggests that the larger PQ particles formed at pH 4 were not associated with improved quercetin entrapment. This observation may once again be attributed to variations in the protein profile—specifically the involvement of collagen—in the soluble fraction obtained following adjustment to pH 4.

**Figure 3:**
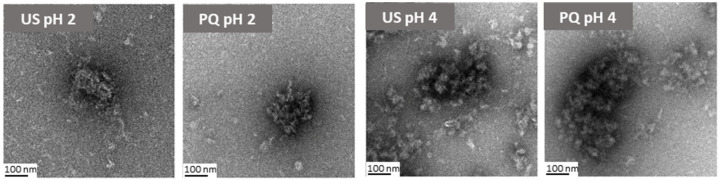
TEM micrographs of ultrasonicated protein (US) and protein-quercetin (PQ) nanoparticles at pH 2 and pH 4.

Based on these results, the acidic PQ dispersions (pH 2 and 4) were selected for further investigation and potential food applications. The morphology of US (ultrasound-treated protein) and PQ (protein–quercetin nanoparticles) at pH 2 and pH 4 was examined using TEM (Figure 3). The US morphology at pH 2 appeared considerably more compact than at pH 4. At the latter pH, numerous small, light structures were observed dispersed throughout the medium, likely corresponding to unbound protein nanoaggregates. This phenomenon was less pronounced at pH 2. The addition of quercetin reduced the prevalence of these presumed free proteins but led to the formation of larger and less spherical nanoparticles at pH 4, consistent with the increased particle size determined by DLS.

### 2.5. Oil-in-Water (O/W) Emulsions

**Figure 4:**
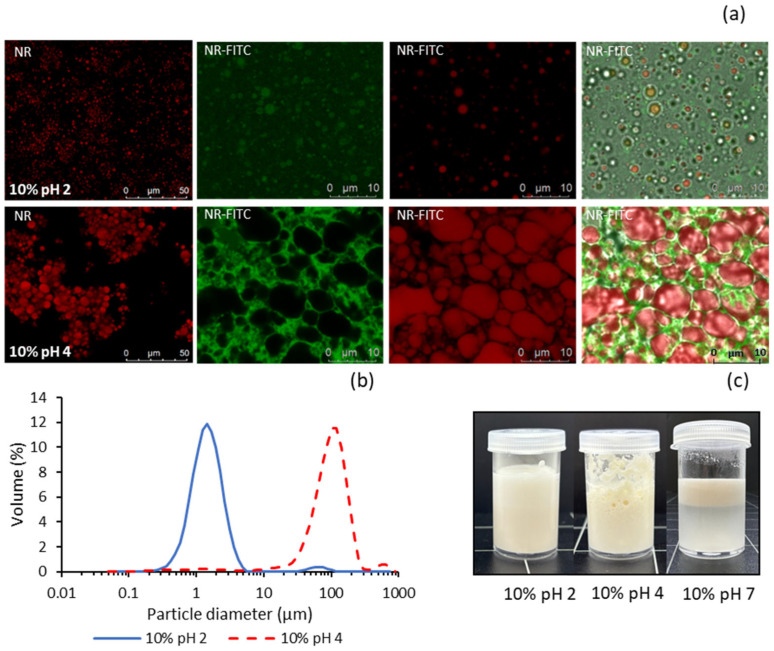
Properties of emulsions made with 10% sunflower oil and PQ dispersions at pH 2 and pH 4: (**a**) CLSM micrographs, (**b**) laser diffraction particle size distribution, (**c**) visual appearance. NR: Nile red, FITC: fluorescein thiocyanate isomer.

PQ dispersions at pH 2 and pH 4 were used to prepare oil-in-water (O/W) emulsions with a low oil content (10%). Both emulsions exhibited a whitish liquid appearance and showed no visible phase separation during at least one week of refrigerated storage (Figure 4c). However, the emulsion at pH 4 tended to form a foamy layer with an aggregated appearance, which was not observed in the emulsion prepared at pH 2. For comparison, an emulsion was also prepared under identical conditions using the PQ dispersion at pH 7. In this case, the emulsion was highly unstable, with complete phase separation occurring in less than 15 min (Figure 4c).

Table 4 presents the apparent viscosity (η_100_) of the emulsions, along with their flow behaviour parameters obtained by fitting the Ostwald–de Waele (power law) model. The pH 7 emulsion was included in the rheological study despite its limited stability. Prior to measurement, it was gently hand-stirred to homogenise the phases before being placed on the rheometer plate. The emulsion at pH 2 exhibited the highest apparent viscosity, whereas the emulsion at pH 7 showed the lowest values (*p* ≤ 0.05). A similar trend was observed in the consistency coefficients (κ_up_ and κ_down_) derived from both the upward and downward shear rate curves. The flow behaviour index (n_up_ and n_down_) decreased in E–pH4 compared with E–pH2, indicating a significant increase (*p* ≤ 0.05) in flow resistance (Table 4). Both emulsions (pH 2 and 4) exhibited pseudoplastic behaviour, as indicated by the power law exponent n < 1 (shear-thinning effect). However, it is noteworthy that n_up_ and n_down_ values were relatively high in both formulations—particularly at pH 2—suggesting a highly fluid character, approaching that of a Newtonian fluid. The highly fluid nature and absence of thixotropy in both emulsions were confirmed by the lack of deviation in κ values between the upward and downward flow curves. Similarly, Pickering emulsions containing 10 wt% oil and stabilised with cellulose nanocrystals have been reported to exhibit liquid-like behaviour [37].

In contrast, the E–pH7 emulsion exhibited a flow index n_up_ > 1 (shear-thickening behaviour) with increasing shear rate, indicating structural build-up during shearing. This effect was accompanied by an increase in κ_down_ relative to κ_up_, as well as n_down_ < 1. These results may be attributed to droplet flocculation or coalescence, along with partial phase separation during analysis, forming large clusters that increase flow resistance. This confirmed the highly unstable nature of the emulsion, indicating that the PQ–pH7 dispersion was not an effective emulsion stabiliser, likely due to insufficient nanoparticle concentration [38]. Particle concentration plays a critical role in determining the stability of Picke-ring emulsions; insufficient particle levels result in incomplete coverage of the oil–water interface, thereby compromising emulsion stability.

Confocal laser scanning microscopy (CLSM) was employed to further compare the pH 2 and pH 4 emulsions, providing insights into how nanoparticle dispersion influences subtle differences in flow properties (Figure 4a). Initially, the emulsions were stained with Nile Red (NR) alone to observe the morphology and distribution of fat droplets, which emitted red fluorescence. Subsequently, both Nile Red and FITC (NR–FITC) were used to differentiate between fat and protein, with the latter emitting green fluorescence. The addition of nanoparticles at pH 2 resulted in much smaller and more uniform fat droplets compared with those at pH 4, where the droplets tended to form larger conglomerates. This effect may be due to the smaller particle size and greater electrostatic repulsion in the dispersion at pH 2, leading to more uniform adsorption of nanoparticles at the oil–water interface. In line with Rajasekaran et al. [15], the markedly elevated protein hydrophobicity observed at pH 2 is likely to facilitate enhanced adsorption of nanoparticles onto the oil droplet interface. The higher particle concentration in the dispersion at pH 2 (94.5% yield) may also contribute to the formation of emulsions with smaller droplet sizes. Similar findings were reported by Zhang et al. [6] using grass carp myofibrillar protein nanoparticles. Furthermore, reduced droplet size has been shown to increase the apparent viscosity of emulsions stabilised by myofibrillar protein nanoparticles, likely due to enhanced inter-droplet interactions [15,16].

When stained with both Nile Red and FITC, the pH 2 emulsion exhibited small spherical green structures attributed to protein nanoparticles adsorbed onto the fat droplets. The aqueous phase background appeared uniformly green, indicating even nanoparticle distribution throughout the dispersion medium. In contrast, the pH 4 emulsion showed larger red droplets embedded in a green gel-like protein network with a significantly higher degree of aggregation. A similar protein network was reported by Zhang et al. [16] using grass carp myofibrillar protein nanoparticles as a stabiliser for Pickering emulsions. This effect may explain the absence of macroscopic phase separation in this emulsion despite the presence of larger, highly agglomerated fat droplets.

Pronounced differences in emulsion droplet size distribution were also observed via DLS (Figure 4b). The D_4,3_ (volume-weighted mean diameter) and D_3,2_ (volume-to-surface area mean diameter) values in the pH 2 emulsion (1.65 ± 0.28 μm and 1.34 ± 0.14 μm, respectively) were significantly smaller than those in the pH 4 emulsion (121 ± 12.6 μm and 13.6 ± 1.27 μm), approaching the nanoscale range. According to CLSM images, the mean diameter of fat droplets in the pH 4 emulsion corresponds to the D_3,2_ value of approximately 14 μm, while the D_4,3_ value of around 120 μm reflects the mean size of droplet aggregates. Based on these observations, the slightly higher viscosity of the pH 2 emulsion can be attributed to its smaller mean droplet size and more uniform, less aggregated microstructure.

## 3. Materials and Methods

### 3.1. Materials

Freshly eviscerated musky octopus (*Eledone moschata*), supplied by Comercial Angulas de Trebujena (Puerto de Santa María, Cádiz, Spain), was transported under refrigeration to ICTAN (Madrid, Spain) within 24 h of capture. The octopus was rinsed with cold tap water, vacuum-packed, and stored frozen at −20 °C until use. Commercial refined sunflower oil (maximum acidity 0.2%) was purchased from El Corte Inglés (Madrid, Spain). Quercetin (≥95%, HPLC grade) was obtained from Sigma-Aldrich (Merck, Darmstadt, Germany). All other reagents were of analytical grade. Pure and ultrapure water from a Milli-Q^®^ Integral Water Purification System was used for all analyses.

### 3.2. Preparation of Protein Concentrate (PC)

Whole specimens of *E. moschata* (around 4 kg) were thawed, and the eyes were manually removed. The specimens were then chopped with crushed ice using a Hobart stainless steel bowl chopper (Model 84181-D, Hobart Corp., Troy, OH, USA) until a uniform blend was achieved. A second homogenisation step was carried out for 2 min at 2 °C using a vacuum homogeniser (Stephan Model FD112M10-72D, Stephan Machinery GmbH, Hameln, Germany). Collagenous residues (primarily derived from remnants of skins and tunics) entangled in the homogeniser helix were manually removed, rinsed under running tap water using a sieve, and subsequently weighed to determine their mass. The minced muscle (M) was mixed with tap water at a 1:2 (*w*/*v*) ratio and solubilised at pH 3.5 using 2 N HCl. This was followed by overnight isoelectric precipitation at approximately pH 5.4 using 1 M NaOH and centrifugation at 12,000× *g* for 40 min at 8 °C in a Sorvall Lynx 6000 centrifuge (Thermo Fisher Scientific Inc., Waltham, MA, USA). The precipitate was frozen without pH adjustment and subsequently lyophilised in a freeze dryer (Model 8KBTEL-85, SP VirTis, Warminster, PA, USA) to obtain the protein concentrate (PC), which was then pulverised, stored at 4 °C under refrigeration, and used within one month, during which the sample consistently yielded reproducible results.

### 3.3. Proximate Analysis and Amino Acid Composition of PC

Moisture and ash contents were determined according to AOAC [39] methods no. 942.05 and no. 950.46, respectively. Total fat was measured following the method of Bligh and Dyer [40]. Total protein was quantified using a LECO-FP 2000 nitrogen/protein analyser (LECO Corp., St. Joseph, MI, USA), applying a nitrogen-to-protein conversion factor of 6.25. The amino acid composition of the protein concentrate (PC) was analysed following acid hydrolysis, as described by Alemán et al. [41]. The nutritional amino acid score (%) was calculated according to FAO/WHO guidelines [30]), using Equation (1):

(1) $$NS\left(\%\right)=\left(\frac{aminoacid\left(mg\;per\;g\;protein\right)}{reference\;value\;WHO(mg\;per\;g\;protein}\right)\times 100$$

### 3.4. Differential Scanning Calorimetry of PC

Differential scanning calorimetry (DSC) was performed on mince (M) and dry protein concentrate (PC) as described by Blanco-Pascual et al. [31]. Briefly, approximately 5 mg of sample was hermetically sealed in aluminium pans, with an empty pan used as the reference. Two consecutive scans were conducted from 5 °C to 90 °C at a heating rate of 10 °C/min, under a dry nitrogen purge (50 mL/min). Endothermic peak temperatures (T_peak, °C) and enthalpies (ΔH, J/g) were analysed using TA Instruments Universal Analysis 2000 software, version 4.5A.

### 3.5. pH Modification and Ultrasonication (US) of PC

The freeze-dried protein concentrate (PC) was dispersed in deionised water (2% *w*/*v*) and magnetically stirred at room temperature for 15 min. The mixtures were adjusted to various pH levels (2 to 10) using 1 M HCl or NaOH, and then homogenised in an Omnimixer for 2 min at 10,000 rpm, with an ice bed to maintain a low temperature. To assess the effect of ultrasonication (US), the samples were sonicated on ice using a probe-tip ultrasonic cell disruptor (Model Q700, Qsonica, Newtown, CT, USA) with a 12.7 mm diameter tip at 50% amplitude for 5 min, employing alternating cycles of 1 min on and 1 min off to prevent overheating. The supernatants, obtained after centrifugation at 10,000× *g* for 20 min at 4 °C, from both non-sonicated (nS) and sonicated (US) protein concentrate dispersions at different pH levels, constituted the soluble protein fractions.

### 3.6. Protein Solubility, Electrophoretic Profile (SDS-PAGE), and Surface Hydrophobicity

The protein concentration in soluble protein fractions from non-sonicated (nS) and ultrasonicated (US) PC was determined using the BCA protein assay kit. Absorbance was measured at 562 nm using a CLARIOstar plate reader (BMG Labtech, Offenburg, Germany). The percentage of protein solubility was calculated based on the protein content in the soluble fraction relative to the total protein content of the PC, previously determined by the Dumas method. All determinations were performed in triplicate.

Electrophoresis (Tris-Glycine-SDS-PAGE) of the nS soluble protein fraction, adjusted to approximately 2 mg/mL protein concentration, was conducted using 10% polyacryl-amide gels (Mini-PROTEAN^®^ TGX™ Precast Protein Gels, Bio-Rad, Madrid, Spain), following the method described by Marín et al. [42]. A molecular weight standard (Precision Plus Protein™ All Blue Prestained Protein Standards, Bio-Rad) was employed.

Surface hydrophobicity (H_0_) was determined in the soluble protein fractions (nS and US) using 8-anilino-1-naphthalenesulfonic acid (ANS) as a fluorescence probe, as described by Lin et al. [27] with slight modifications. Fluorescence intensity (FI) was measured using an excitation wavelength of 375 nm and an emission wavelength of 480 nm in the CLARIOstar plate reader. Surface hydrophobicity (H_0_) was expressed as the slope of the line obtained from linear regression of FI against protein concentration (mg/mL). All determinations were performed in triplicate.

### 3.7. Complexation of Quercetin with US-Treated PC

Quercetin (1% *w*/*v*) was dissolved in absolute ethanol. A 2 mL aliquot of the quercetin solution was added dropwise to 50 mL of the protein dispersion (2% *w*/*v*), which had previously been adjusted to various pH levels and ultrasonicated as described above. The samples were stirred magnetically for 1 h at room temperature, then centrifuged at 10,000× *g* for 20 min at 4 °C. Protein–quercetin (PQ) nanoparticles were collected in the supernatants. The precipitates were used to evaluate the yield gravimetrically after drying in an oven at 105 °C. The yield (%) was calculated using Equation (2):

(2) Y=100−(Pf∗100/P0)

where Pf is the weight of the dried precipitate and P0 is the initial weight of dry protein concentrate and quercetin used to produce the PQ nanoparticles.

The entrapment efficiency (EE, %) of quercetin was determined following the method of Carrasco-Sandoval et al. [43], with slight modifications. Ethyl acetate (1:3, *v*/*v*) was employed to extract quercetin from the PQ dispersions. After vortexing for 10 s, the mixture was centrifuged at 10,000× *g* for 20 min to fully separate the precipitated nanoparticles from the non-entrapped quercetin present in the organic phase. The ethyl acetate layer was collected and evaporated to dryness under a nitrogen stream. The residue was reconstituted in 2 mL of methanol and filtered through a 0.22 μm syringe filter. Quercetin content was quantified by RP-HPLC as previously described by Alemán et al. [44]. Entrapment efficiency (EE) was calculated using Equation (3):

(3) $$\%EE=\frac{entrapped\;quercetin}{total\;quercetin}\times 100$$

The amount of entrapped quercetin was calculated as the difference between the total quercetin used for nanoparticle preparation and the non-entrapped quercetin recovered.

### 3.8. Preparation of Oil-in-Water Emulsions

Commercial sunflower oil was homogenised with PQ nanoparticle dispersions at pH 2 and 4 (aqueous phase) in an oil-to-water ratio of 10:90 (*v*/*v*), using an Ultra-Turrax T25 homogeniser (IKA-Werke GmbH, Staufen, Germany) at 12,000 rpm for 1 min. A subsequent ultrasonication treatment was applied for 5 min at 50% amplitude, employing alternating cycles of 1 min on and 1 min off. An emulsion with a 10:90 ratio containing PQ at pH 7 was also prepared under identical conditions.

### 3.9. Characterisation of Particle Size and ζ Potential

The mean particle size (z-average, nm) and ζ potential (mV) of soluble protein fractions (nS and US) and PQ nanoparticle dispersions were determined by Dynamic Light Scattering (DLS) and Electrophoretic Light Scattering (ELS), respectively, in triplicate, using a Zetasizer Nano ZS particle analyser (Malvern Instruments Ltd., Worcestershire, UK), following appropriate dilution in deionised water at 25 °C.

Mean particle size in the emulsions was also determined in triplicate using laser diffraction analysis with a Malvern Mastersizer 2000 particle size analyser. The De Brouckere mean diameter (D_4,3_) and Sauter mean diameter (D_3,2_) were calculated using the software provided with the instrument.

### 3.10. Transmission Electron Microscopy

The morphology of ultrasonicated protein dispersion (US) and protein–quercetin (PQ) nanoparticles at pH 2 and 4 was examined using transmission electron microscopy (TEM) with a Thermo Fisher TALOS L120C microscope operated at 120 kV. Samples were analysed after adsorption onto carbon-coated grids subjected to glow discharge and stained with 2% uranyl acetate. Images were captured using a Thermo Fisher CETA-F camera.

### 3.11. Rheological Properties

The flow behaviour of the emulsions was assessed at 25 °C using a Discovery H10 rheometer (TA Instruments, Waters Corporation, New Castle, DE, USA) equipped with cone–plate geometry, as described by Pascual-Silva et al. [45]. Shear rate ramps were applied from 0.1 s^−1^ to 100 s^−1^ and back to 0.1 s^−1^, with a pre-shear step at 100 s^−1^ for 20 s in each case. Viscosity as a function of shear rate (s^−1^) was plotted for both the upward and downward ramps. Apparent viscosity (η_100_) values were obtained from the initial pre-shear step at 100 s^−1^. The Ostwald–de Waele (power law) model, based on shear stress versus shear rate curves, was applied according to Equation (4):

(4) $$\tau =\kappa \cdot \gamma^{n}$$

where τ represents the shear stress, κ is the flow consistency index, γ is the shear rate and n is the flow behaviour index. Thixotropy was evaluated from the hysteresis loop between the upward and downward curves using HR10 TA Instruments Trios software.

### 3.12. Confocal Laser Scanning Microscopy (CLSM)

Emulsions prepared with ultrasonicated protein dispersion (US) and protein–quercetin (PQ) nanoparticles at pH 2 and 4 were examined using confocal laser scanning microscopy (CLSM) with a LEICA TCS SP5 microscope equipped with a Hamamatsu Orca Flash 4.0 LT camera. Nile red, dissolved at 0.01% in ethanol, and fluorescein isothiocyanate isomer (FITC), dissolved at 1 mg/mL in DMSO and subsequently diluted to 0.01% in deionised water, were used to visualise fat droplets (red signal, emission range 618–639 nm) and protein particles (green signal, emission range 536–585 nm), respectively. Approximately 10 μL of each fluorophore was added to the emulsions, which were then placed on optical glass slides for analysis.

### 3.13. Statistical Analysis

A one-way analysis of variance (ANOVA) was performed using SPSS^®^ software (IBM SPSS Statistics v29, Chicago, IL, USA). Differences between means were assessed using confidence intervals and Tukey’s range test, with statistical significance set at *p* ≤ 0.05. All determinations were performed in triplicate (n = 3), and the results are presented as mean ± standard deviation.

## 4. Conclusions

A marine species of low commercial value was valorised through the production of a protein-rich concentrate via acid solubilisation and isoelectric precipitation. Pretreatment of the protein concentrate by dispersing it in water at varying pH levels, followed by ultrasonication, significantly affected the particle characteristics, yield and quercetin entrapment efficiency of the resulting protein–quercetin nanoparticles. These effects were attributed to differences in protein solubility, electrophoretic profile, and hydrophobicity. Dispersing the lyophilised protein concentrate at acidic pH levels (2 and 4) proved particularly effective in terms of particle morphology, yield, and quercetin entrapment efficiency. Notably, the electrophoretic protein profile indicated a substantial contribution of soluble collagen in the sample dispersed at pH 4. Compared with pH 7, nanoparticle dispersions at acidic pH effectively suppressed phase separation in low-fat Pickering emulsions, which also exhibited pronounced fluidity in their flow behaviour. Microscopically, the lower apparent viscosity observed at pH 4 was attributed to a less uniform and more aggregated microstructure. Both acidic emulsions show promise for enhancing the visual appeal and palatability of liquid functional foods—such as vegetable smoothies, light creams, and salad dressings—while offering a low-calorie alternative. Although the present study was designed as a fundamental investigation, the findings provide an early indication of the system’s potential for industrially relevant applications. Because the preparation relies solely on simple unit operations—pH shifting, ultrasonication, and centrifugation—the process can be readily integrated into existing food-processing workflows. Overall, these results suggest that, with further optimisation and techno-economic assessment, the proposed system could offer considerable value for practical industrial deployment.

## Figures and Tables

**Table 1:** Proximate composition of musky octopus mince (M) and freeze-dried protein concentrate (PC).

	Mince (M)	Protein Concentrate (PC)
Moisture (%)	82.83 ± 0.77	2.41 ± 0.43
Protein (%)	15.14 ± 0.23	89.63 ± 0.04
Ash (%)	1.20 ± 0.19	3.04 ± 0.05
Fat (%)	0.62 ± 0.13	4.86 ± 0.08

**Table 2:** Amino acid composition of the freeze-dried protein concentrate (PC).

	mg/g Protein	Nutritional Score (%)	FAO/WHO Reference Pattern (mg/g) ^1^
Aspartic acid	100.49 ± 0.34		
Threonine	48.57 ± 0.35	211.17	23
Serine	50.22 ± 0.84		
Glutamic acid	148.28 ± 0.87		
Glycine	53.32 ± 0.08		
Alanine	50.69 ± 0.16		
Cysteine	5.97 ± 0.55		
Valine	43.46 ± 0.62	111.44	39
Methionine	32.66 ± 2.33		
Isoleucine	48.18 ± 0.43	160.60	30
Leucine	78.80 ± 0.18	133.56	59
Tyrosine	39.99 ± 0.38		
Phenylalanine	43.32 ± 0.15		
Hydroxylysine	3.87 ± 0.03		
Histidine	23.44 ± 0.25	156.27	15
Lysine	76.70 ± 0.44	170.44	45
Arginine	83.25 ± 0.77		
Hydroxyproline	12.14 ± 0.10		
Proline	56.64 ± 0.32		
∑ Hydrophobic	407.13 ± 3.73		
∑ TEAA ^2^	395.06 ± 2.50		
Met + Cys	38.64 ± 2.88	175.59	22
Phe + Tyr	83.31 ± 0.53	219.24	38

^1^ [30]; ^2^ Total essential amino acids.

**Table 3:** Particle yield and entrapment efficiency (EE) of protein-quercetin nanoparticles at various pH levels.

	Yield (%)	Entrapment Efficiency (%)
PQ-pH 2	94.5 ± 2.2 ^d^	81.2 ± 1.2 ^d^
PQ-pH 4	72.2 ± 9.3 ^c^	57.2 ± 5.1 ^b^
PQ-pH 7	24.2 ± 1.6 ^a^	65.7 ± 1.9 ^c^
PQ-pH 8	36.6 ± 1.7 ^b^	39.9 ± 3.8 ^a^
PQ-pH 10	92.5 ± 1.7 ^d^	81.9 ± 1.0 ^d^

Different letters (a, b, c…) indicate significant differences (*p* ≤ 0.05).

**Table 4:** Apparent viscosity and flow parameters (derived from Equation (4), including upward and downward flow curves) of emulsions prepared with 10% oil and PQ dispersions at pH 2, 4, and 7.

	Apparent Viscosity η_100_ (mPa∙s)	κ_up_ (mPa⋅s^n^)	κ_down_ (mPa⋅s^n^)	n_up_	n_down_
PQ-pH 2	8.42 ± 0.09 ^a^	8.87 ± 1.41 ^a^	8.88 ± 3.75 ^a^	0.938 ± 0.002 ^a^	0.944 ± 0.061 ^a^
PQ-pH 4	2.26 ± 0.03 ^b^	4.52 ± 0.46 ^b^	4.51 ± 0.19 ^b^	0.864 ± 0.023 ^b^	0.863 ± 0.011 ^b^
PQ-pH 7	1.88 ± 0.08 ^c^	0.93 ± 0.12 ^c^	2.73 ± 0.06 ^b^	1.180 ± 0.024 ^c^	0.878 ± 0.034 ^ab^

Different letters (a, b, c) indicate significant differences (*p* ≤ 0.05).

## Data Availability

Dataset available on request from the authors.
